# Vertigo and Nystagmus: The Role of the Velocity Storage Integrator in Skull Vibration Induced Nystagmus (SVIN)

**DOI:** 10.3390/audiolres16050156

**Published:** 2026-10-09

**Authors:** Ian S. Curthoys

**Affiliations:** Vestibular Research Laboratory, School of Psychology, University of Sydney, Sydney, NSW 2006, Australia; ian.curthoys@sydney.edu.au

**Keywords:** vertigo, nystagmus, semicircular canal, otolith

## Abstract

This paper reviews the vestibular neural mechanisms that generate vertigo and nystagmus. Angular acceleration activates a group of neurons in the brainstem that cause the person to have the sensation of turning. But long after the end of the acceleration, these neurons continue to fire; they perseverate, causing afternystagmus, and I contend that this gives the false sensation that the person is continuing to turn when they are actually stationary. That false sensation, that illusion, is called vertigo, and the neural basis for it is the continued firing of this cluster of neurons in the brainstem which signal turning—firing long after the turn has stopped. Any other process that generates activity of this group of neurons—disease, dysfunction, other sensory input—will also result in that false sensation of turning—vertigo. The group of neurons is called the velocity storage integrator (VSI). The operation of this VSI network is directly linked to neural systems controlling nausea and postural stability, and it is a key part of a neural circuit generating the subjective sensation of vertigo. The VSI receives input from vestibular, optokinetic, and proprioceptive systems, and it projects to many neural systems to generate nystagmus, sensation, nausea, and postural responses.

## 1. Introduction

This paper reviews evidence about neural activation in response to angular acceleration and skull vibration and, using data from animal experiments, puts forward a hypothesis linking activity of a group of brainstem neurons called the velocity storage integrator and the human subjective sensation of vertigo.

Over time after the end of a horizontal (yaw) angular acceleration, both the subjective sensation of turning—the vertigo—and the afternystagmus both decline in close correspondence until the person again feels stable. I use the term vertigo to refer to this fundamentally important “illusion”: the person is physically stationary yet experiences an overwhelming sensation of turning. This is what patients with vestibular disorders report—that while seated stationary, they feel as though they are turning. Their sense of self-stability has been disrupted by the vestibular disorder. There are wide varieties of vertigo [1] but I will use the term vertigo in the present paper as defined above. Accordingly, understanding the neural basis of vertigo and its relationship to nystagmus is directly relevant to understanding the experience of patients.

Angular acceleration and linear acceleration produce a sustained deflection of the hair bundles of vestibular receptors—in the canals via deflection of the cupula, in the otoliths via deflection of the otolithic membrane. These sustained deflections of the type I and type II receptor hair cells cause sustained increases in firing of afferent neurons, resulting in the vestibulocochlear response [2]. In primary afferents there is a wide range of the regularity of action potential firing at rest, but a useful simplification is to divide afferents into two major classes of afferent neurons according to the regularity of their resting discharge—regular and irregular. There are many differences in the responses of regular and irregular afferents to vestibular stimuli. Both classes are activated by deflection of the cupula or otolithic membrane, but, as will be shown below, it is only irregular afferents that are activated by sound or vibration. It is important not to try to link the regularity of discharge directly to the two types of receptor hair cells, type I and type II, because many regular, tonic afferents and many irregular, adapting afferents get input from both type I and type II hair cells [3,4,5]. Because of this input from both receptor types onto both regular and irregular afferents, it is simply not possible to link afferent regularity to receptor types, and in this paper, the results of afferent neurons are reviewed.

**Figure 1:**
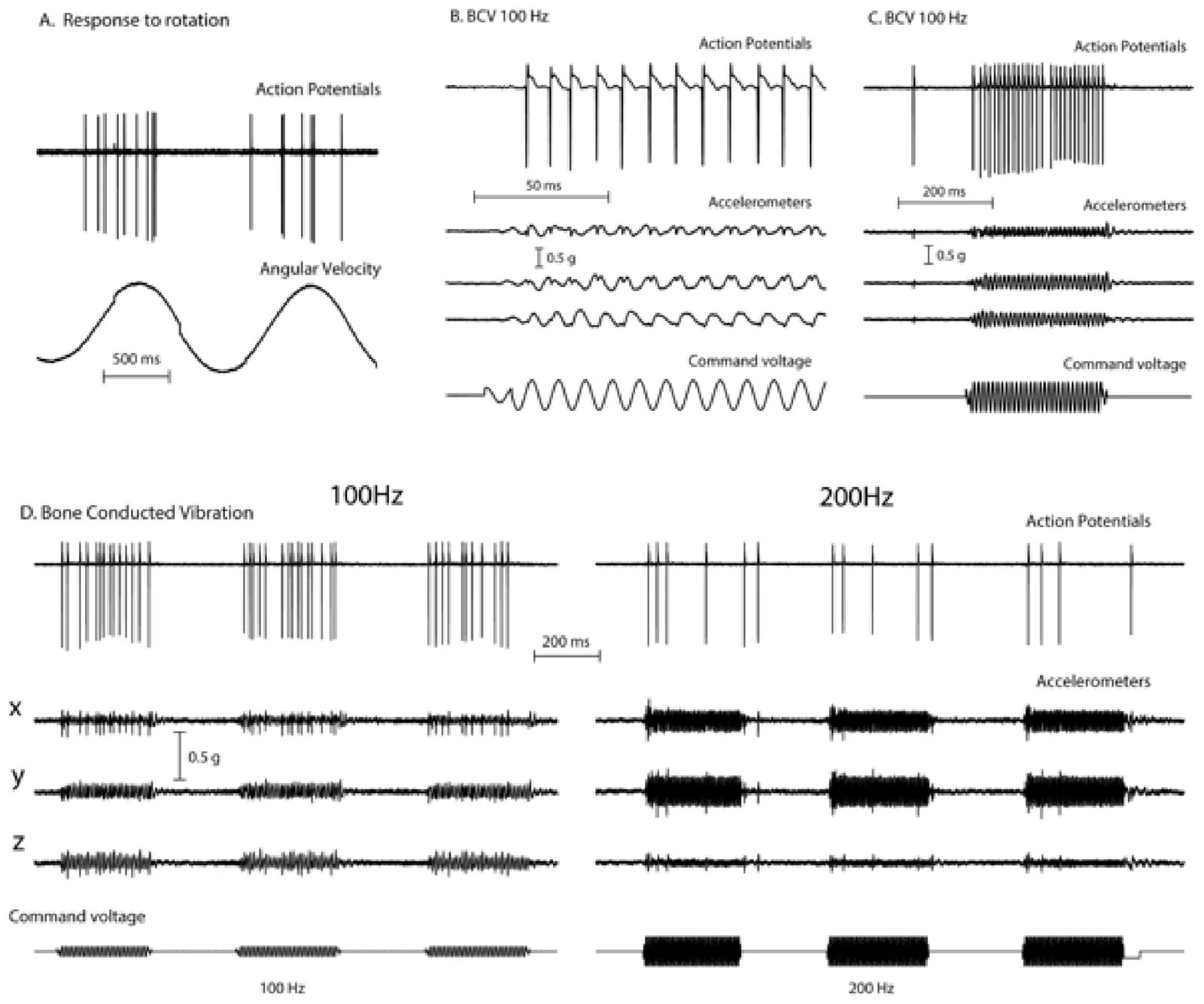
The responses (action potentials) of an irregular primary semicircular canal neuron to 100 Hz skull vibration. (**A**) Responses to angular acceleration, demonstrating that this is a semicircular canal neuron. (**B**) In response to 100 Hz skull vibration, the neuron fires one action potential per cycle, and it is phase-locked to the stimulus waveform, as shown by the small spikes in the accelerometer records and the very even spacing of action potentials in (**C**). However, if frequency is increased, the neural response decreases (**D**), even when the vibration intensity is increased (as shown by the smaller neural response at 200 Hz compared to 100 Hz). Reproduced from [7] with permission of Wolters Kluwer Health.

Vibration-driven receptor activation of vestibular receptors and irregular afferents contrasts with the usual mode of canal activation via cupula deflection. With vibration, the stimulus transmitted through bone and fluid is sufficient to deflect receptor hair cells and so activate afferent neurons once per cycle, which is totally different from the maintained hair bundle deflection caused by the usual vestibular responses to acceleration stimuli. The great sensitivity of vestibular hair cells (with activation thresholds around 10 s for nanometers of deflection [6] means that such an unconventional vestibular stimulus as vibration can nonetheless trigger a full vestibular response, with every single cycle of 100 Hz vibration generating a synchronized action potential, as shown by single-unit recordings from identified irregular primary canal and otolith afferents during 100 Hz vibration [7,8] (Figure 1). In response to vibration, canal and irregular afferent neurons show action potentials synchronized to stimulus frequency. This is called phase locking, and it is very well understood for auditory afferents [9,10] but it applies to vestibular afferents as well [11,12].

Recordings of single primary vestibular afferent neurons in anaesthetized guinea pigs show that 100 Hz vibration selectively activates primary vestibular afferents with irregular resting discharge, in both the canal and otolith systems. 100 Hz vibration is not effective in activating afferent neurons with regular resting discharge [13]. Irregular afferents innervate receptors at the striola of the otolithic maculae and the crest of the crista, where they synapse on type I hair cells via calyx or dimorphic endings. However, skull vibration is a unique stimulus in that it causes simultaneous synchronized activation of irregular canal and otolith afferents.

**Figure 2:**
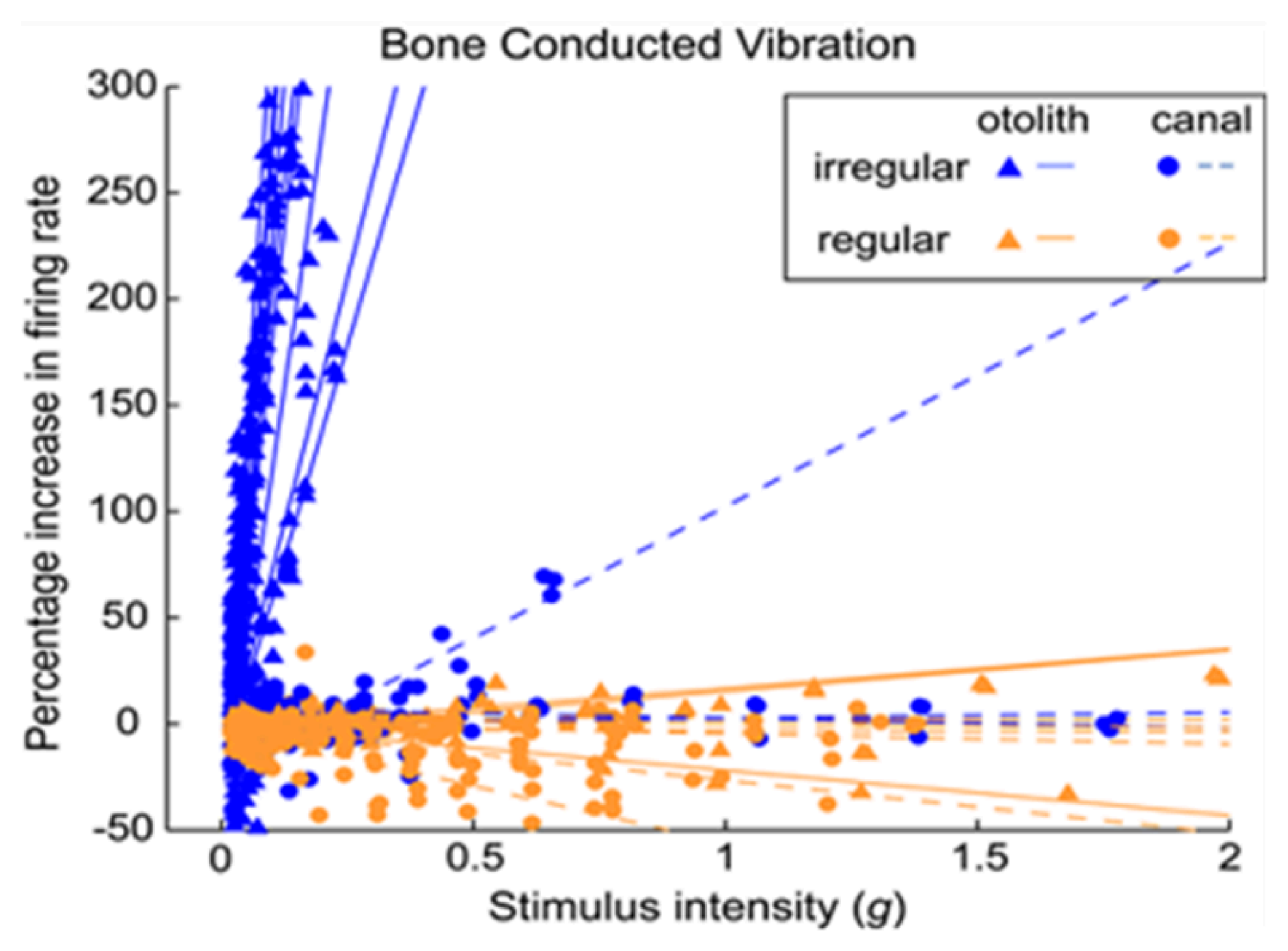
Plots of the changes in firing rate with increasing stimulus intensity for irregular neurons (blue) and regular neurons (orange). Each line is the linear fit to the increase in firing in response to increased stimulus magnitude for each different single neuron. Irregular neurons are activated at very low intensities and have a very sensitive response to increasing intensity. In contrast, regular neurons do not respond even up to stimulus intensities of 2 g. Reproduced from [18] with permission.

In the otoliths, the hair bundles of receptors at this special region of the otolithic macula called the striola are short and have comparatively tenuous coupling to the utricular membrane [14,15,16]. It is irregular afferent neurons from this region [17] which are especially responsive to sound and vibration [18]. Goldberg showed that irregular otolithic afferents terminate on type I striolar receptors [19]. That result was complemented by data from guinea pigs showing that irregular otolithic afferents are responsive to sound and vibration [18,20] and have calyx and dimorphic termination on striolar receptors (reviewed in [18]). Extrastriolar receptors have longer cilia tightly coupled to the otolithic membrane, are thus less likely to respond to sound [16] and it is afferents from extrastriolar areas which have regular resting discharge [19]. Otolith afferents with regular resting discharge are rarely activated by vibration [20] (Figure 2). Irregular canal afferents also apparently preferentially originate from the crest of the crista [21,22], and it is this location where irregular canal afferents activated by vibration originate from [7]. This differential activation of irregular and regular afferents to vibration stimulation presents an opportunity to identify the relative contributions of irregular and regular primary vestibular afferents to the velocity storage integrator at the brainstem and thus their relative contributions to vestibular function, including vertigo.

Vestibular receptors are highly sensitive, and deflections of hair bundles of the order of only tens of nanometres are sufficient to activate them allowing irregular primary canal afferents to fire once per vibration cycle—phase-locked—at frequencies up to about 200–300 Hz. Phase locking applies to irregular afferents in both the otolith and canal systems. The phase locking of otolith irregular afferents still holds for frequencies far higher—beyond 1000 Hz—although there is not an action potential on each cycle; however, synchronization holds because exactly when the afferent fires is locked to the stimulus [12]. This is analogous to the phase locking of auditory afferents to sound [9,23]. Phase-locked activation is a unique characteristic of vestibular neurons with irregular resting discharge, since afferents with regular resting discharge are rarely activated by sound or vibration at clinically relevant stimulus levels [20] (see Figure 2).

In summary: The cycle-by-cycle activation of irregular afferents is fundamentally different from the sustained activation produced by acceleration or caloric stimulation. At frequencies such as 100 Hz, canal and otolith irregular afferents are activated simultaneously on each cycle. This means 100 Hz sound or vibration is unusual—it causes simultaneous activation of both canal and otolith irregular afferents.

## 2. How Is This Information Transmitted from the Vestibular Receptors to the Brain

“Experimental evidence and modelling indicate that there are two parallel pathways, called the direct and indirect pathways, which transmit neural information from the vestibular receptors in the canals and otoliths to central mechanisms. The direct pathway consists of the fast three-neuron arc between the vestibular receptors and the ocular motoneurons and is responsible for the fast compensatory eye movements to head accelerations. The slower indirect pathway has very different response characteristics: In response to a step of velocity, it holds or “stores” activity from the vestibular periphery and discharges it over a more prolonged timespan to produce eye velocity against head velocity; hence the name “velocity storage” and the designation “indirect” in modelling terms” [24] p. 155. The indirect pathway includes the velocity storage integrator that generates vestibular responses—oculomotor, postural, autonomic, and sensory responses, including self-motion, self-stability, and vertigo. Both pathways are activated by acceleration, but the two pathways have very different characteristics and functions. The two pathways can be dissociated—selective lesion of the midline of the brainstem eliminates afternystagmus (the unique indicator of the indirect pathway [25,26]) but the direct pathway remains [27].

I contend that the **direct** pathway consists of large-diameter afferent neurons with irregular resting discharge [28,29]. These neurons mediate fast, short-latency compensatory responses such as the compensatory eye movement response seen in the very fast head impulse test of semicircular canal function, and the short-latency myogenic response to transient otolithic stimulation (vestibulo-ocular evoked myogenic potentials, VEMPs). It is my contention that it is these irregular afferent neurons from the canals and otoliths forming the direct pathway which are activated by vibration [13].

In contrast, I contend that the **indirect** pathway consists of small-diameter afferents with regular resting discharge, which project to the velocity storage integrator. As I show below, this network is responsible for afternystagmus and the sensation of self-motion and vertigo. The VSI is not exclusively vestibular—the network integrates vestibular input with other sensory input (visual optokinetic input, somatosensory input, (see below) and is probably responsible for the sensation of turning and the prolonged afternystagmus and vertigo that follows. Activation of these regular afferents results in nystagmus and afternystagmus as I now discuss.

## 3. Skull Vibration Induced Nystagmus (SVIN)

**Figure 3:**
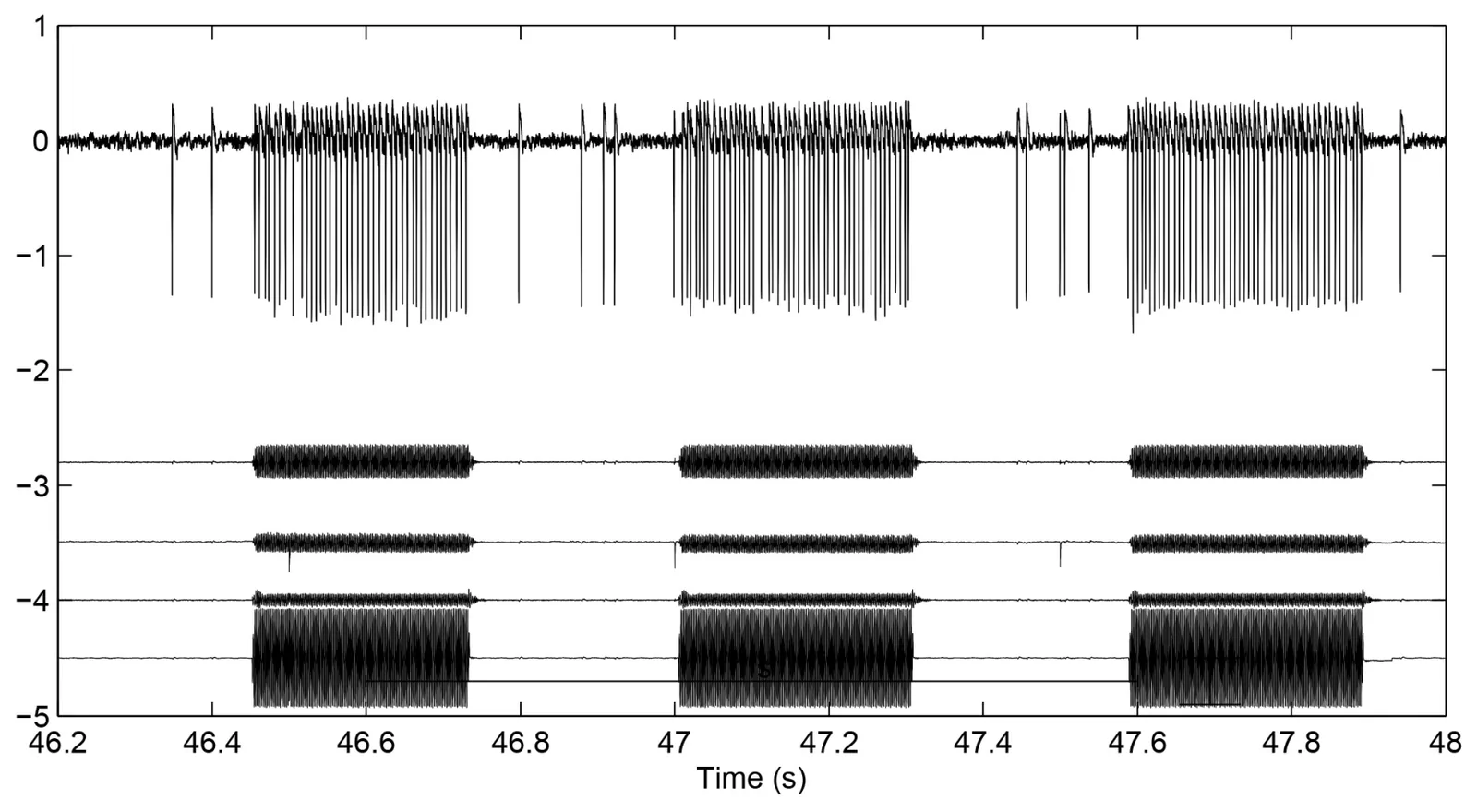
Action potentials of a single irregular semicircular canal neuron in response to three bursts of 500 Hz bone-conducted vibration, showing the neuron’s phase-locked activation is itself tightly locked to the presentation of the stimulus. These show the abrupt onset and offset of the neural activation without any after-discharge. The records show the action potentials and then 3 accelerometer traces and the command voltage. Replotted from data published in [32].

**Figure 4:**
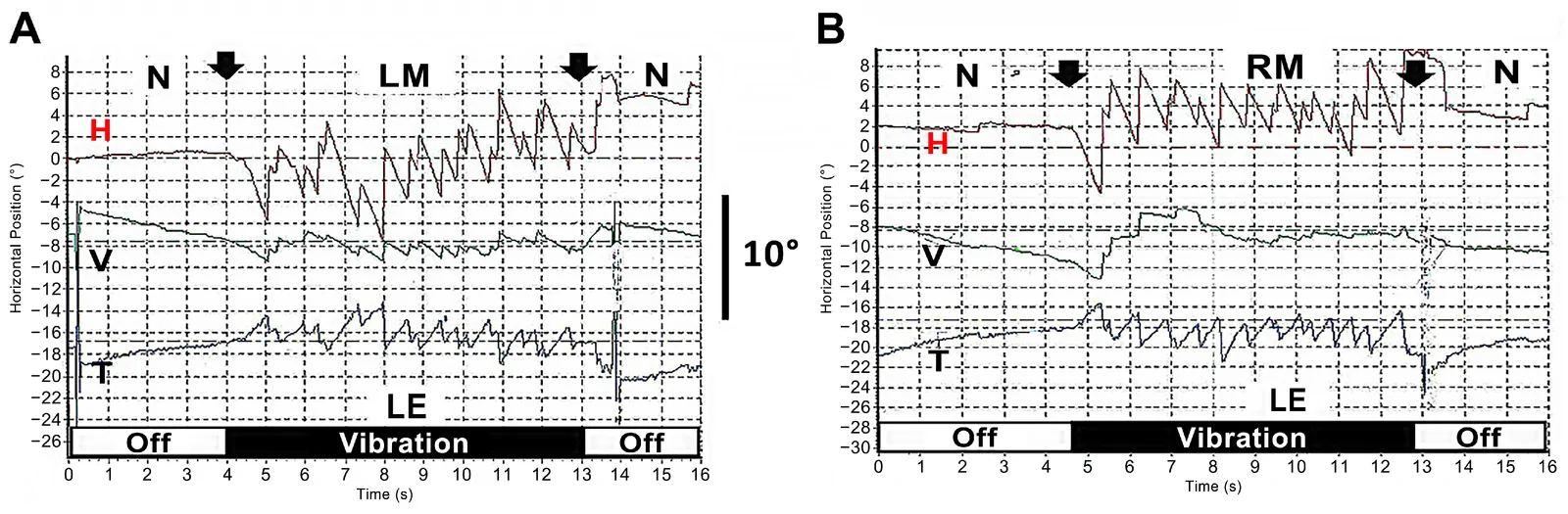
Examples of skull vibration-induced nystagmus in response to 100 Hz vibration stimulation of each mastoid of a patient with complete unilateral vestibular loss. (**A**) left mastoid. (**B**) right mastoid. H horizontal, V vertical, T torsional. RM: right mastoid, LM: left mastoid. The skull vibration-induced nystagmus is the same direction for stimulation of both left and right mastoids and has an abrupt onset at full slow-phase eye velocity with stimulus onset and is maintained during this stimulus and abruptly ceases at the end of the stimulus, with no afternystagmus. This pattern of nystagmus corresponds closely to the way in which neural activation of irregular primary neurons is tightly coupled to the vibration stimulus (Figure 3). Reproduced with permission from [33].

As the physiological evidence about the effect of vibration on vestibular receptors and afferents was building up (reviewed in [18] so the evidence about the effect of mastoid vibration on nystagmus in human patients was also developing (reviewed in [30]). In darkness, the application of 100 Hz vibration to either mastoid of a patient with complete unilateral vestibular loss produces predominantly horizontal nystagmus with quick phases beating away from the affected ear. This is Skull Vibration Induced Nystagmus (SVIN). Note that partial vestibular loss is less definitive. It is my contention that the nystagmus arises from activation of vestibular receptors and irregular afferents by the vibration stimulus, because the onset and offset of the nystagmus are so tightly locked to the onset and offset of vibration stimulation, just as the activation of irregular primary afferents is similarly tightly stimulus-locked to stimulus onset and offset (see Figure 3 [14,31] and compare to human nystagmus response to 100Hz skull vibration Figure 4).

In patients with unilateral vestibular loss, the quick phases of SVIN beat away from the affected ear regardless of which mastoid is stimulated [30], probably because vibration is so effectively transmitted through the skull that it is a stimulus for both peripheral vestibular systems. SVIN is optimally elicited by 100 Hz vibration reference: Dumas). Unlike caloric or rotational nystagmus, SVIN begins at full slow-phase velocity at the very onset of the vibration stimulus, and usually shows little adaptation during the stimulus, and ceases abruptly at vibration offset—with very little or no afternystagmus (Figure 4). Healthy individuals, with both vestibular systems functioning normally, do not show SVIN above a clinically defined threshold of slow phase eye velocity of 2.5 deg/s, presumably because both labyrinths are stimulated simultaneously and roughly equally, so their neural input probably effectively cancels at the vestibular nuclei [34]. Such cancellation with simultaneous bilateral stimulation has previously been demonstrated using high-frequency electrical stimulation of both labyrinths in cats [35]. In patients with unilateral vestibular loss, however, vibration-driven neural activity from the intact side is no longer cancelled by simultaneous input from the affected (non-functional) side. So, in the patient with unilateral vestibular loss, the resulting imbalance between the two vestibular nuclei generates nystagmus, in the same way that acute unilateral loss or caloric stimulation produces a nystagmus driven by the vestibular nuclei imbalance [34]. So, the presence of SVIN indicates asymmetrical vestibular function between the two vestibular nuclei.

Since the vibration causes phase-locking of irregular afferent neurons, at frequencies like 30 Hz there is an action potential generated on every single cycle of the vibration stimulus. This has the interesting consequence that increasing vibration frequency increases neural firing rate. In correspondence, nystagmus slow-phase velocity increases as vibration frequency is increased, so that the slow-phase eye velocity to vibration at 100 Hz is significantly above the slow-phase velocity at 30 Hz [36]. 100 Hz is optimal for producing SVIN in human patients; lower frequencies produce nystagmus of lower slow-phase velocity corresponding to the lower synchronized firing rates such a stimulus evokes in irregular vestibular afferents. While vibration at frequencies above roughly 150 Hz becomes progressively less effective at activating canal neurons (see Figure 1), these stimuli remain effective stimuli for otolithic neurons. So, 500 Hz mastoid vibration, for instance, activates otolithic receptors and afferents but does not activate canal receptors and neurons and so does not usually evoke SVIN. After SCD, skull vibration activates neurons at frequencies beyond 1000 Hz, and in contrast, patients with SCD at high vibration frequencies can elicit SVIN [36].

The very different characteristics of SVIN—its immediate onset, absence of build-up, and abrupt cessation—set it apart from caloric or nystagmus to angular acceleration, both of which responses build up gradually as the cupula is deflected. and both of which decay slowly after stimulus offset as the cupula returns to its resting position. This is because angular acceleration causes cupula deflection and produces a progressive increase in afferent firing and corresponding increase in the slow phase eye velocity of nystagmus. In contrast, the very first cycle of a 100 Hz mastoid vibration generates an action potential, as does each succeeding cycle—resulting in a constant firing rate of irregular afferents (see Figure 1) during the vibration stimulus, and the nystagmus shows a corresponding constant slow phase velocity during the vibration stimulus (see Figure 4B).

## 4. Why Is Afternystagmus Important?

**Figure 5:**
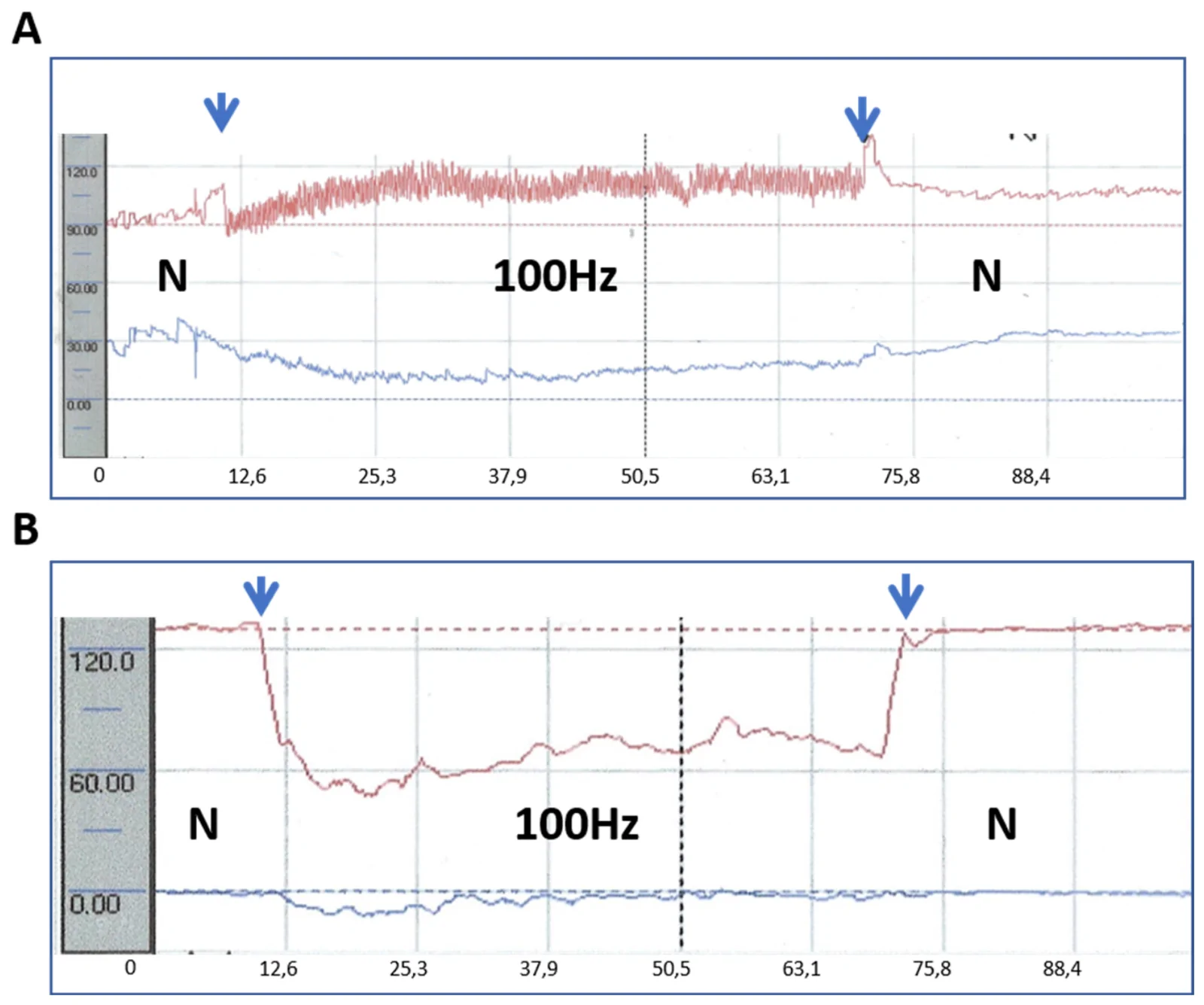
SVIN in a patient with unilateral vestibular loss in response to long-duration 100 Hz stimulation. There is an abrupt onset and offset of SVIN (downward arrows) with little or no afternystagmus or vertigo even after prolonged stimulation. (**A**) Eye position records for horizontal (upper record) and vertical (lower record). (**B**) Corresponding eye velocity measures, showing minimal horizontal afternystagmus after 60 s skull vibration nystagmus. Reproduced from [37] with permission.

Afternystagmus is the term I use to describe the nystagmus that continues after the end of the stimulus. For angular acceleration stimuli, the initial part of afternystagmus is due to cupula return, but in healthy subjects (and monkeys), that afternystagmus continues for many seconds after the cupula has returned to its resting position [25,26]. In contrast, in patients with complete unilateral vestibular loss given a 10 s vibration stimulus, SVIN continuing after the termination of the stimulus is rarely observed and, when present, is minimal (Figure 4B). Even with 60 s vibration stimulation—SVIN persists throughout the vibration and ceases abruptly at offset, and there is usually no meaningful afternystagmus (Figure 5). This patient had minimal afternystagmus after the end of the 60 s 100 Hz skull vibration stimulation and reported no sensation of vertigo after the end of the 60 s vibration.

The nystagmus in response to skull vibration is clearly caused by completely different means compared to the nystagmus caused by angular acceleration. In summary, vibration does not usually cause afternystagmus, whereas angular acceleration does.

## 5. How Does the Velocity Storage Integrator Work?

There are two separate neural integrators associated with oculomotor function. One is related to gaze holding, being responsible for the “velocity-to-position” conversion that occurs when ocular motoneurons are activated from the vestibular system [38,39]. This integrator is used to hold positions of fixation associated with saccades [40]. The second integrator is related to maintenance of slow phase eye and head (gaze) velocity through velocity storage [41]. The present paper is focused on eye velocity in nystagmus, not eye position, and the integrator referred to below for these data is the velocity storage integrator, not the velocity-to-position integrator.

It is useful to think of the velocity storage integrator as being comprised of two rather different parts. One part—the nystagmus generator—generates the slow and quick phases of nystagmus; the other part—the velocity storage integrator—which is, I contend, a major part of the neural network responsible for vertigo, is held to be responsible for enhancing the oculomotor response to low-frequency head rotations. From its inception, the marker of velocity storage has been afternystagmus. The unique nystagmus response to vibration stimulation allows these two parts to be dissociated.

## 6. The Nystagmus Generator

Based on extensive evidence, it is my contention that irregular vestibular afferents in the direct pathway project predominantly to a subgroup of brainstem burst and burst-tonic neurons which constitute what I term the nystagmus generator subgroup of the VSI network [42]. The neurons in this subgroup in turn drive neurons in the abducens nucleus to produce the characteristic slow and quick-phase pattern of eye movements which constitute nystagmus [43]. The neurons in this subgroup are called VPS neurons since these neurons are activated by vestibular stimuli but cease firing at the nystagmus quick phase, and so the firing pattern is [vestibular +pause]. I contend that VPS neurons form the nystagmus generator subgroup, and they are located close to the midline and close to the prepositus hypoglossi, projecting to the abducens nucleus as shown by Shimazu and colleagues, [44] and that they receive direct input from irregular afferents. Whereas I contend that regular afferents project to vestibular brainstem neurons which do not pause and are referred to as vestibular only or VO neurons. (Neurons in the nystagmus generator are also known to have their activity suppressed by visual stimuli, likely via cerebellar inhibition [45]; this may explain why SVIN is itself suppressed by vision [46]).

**Figure 6:**
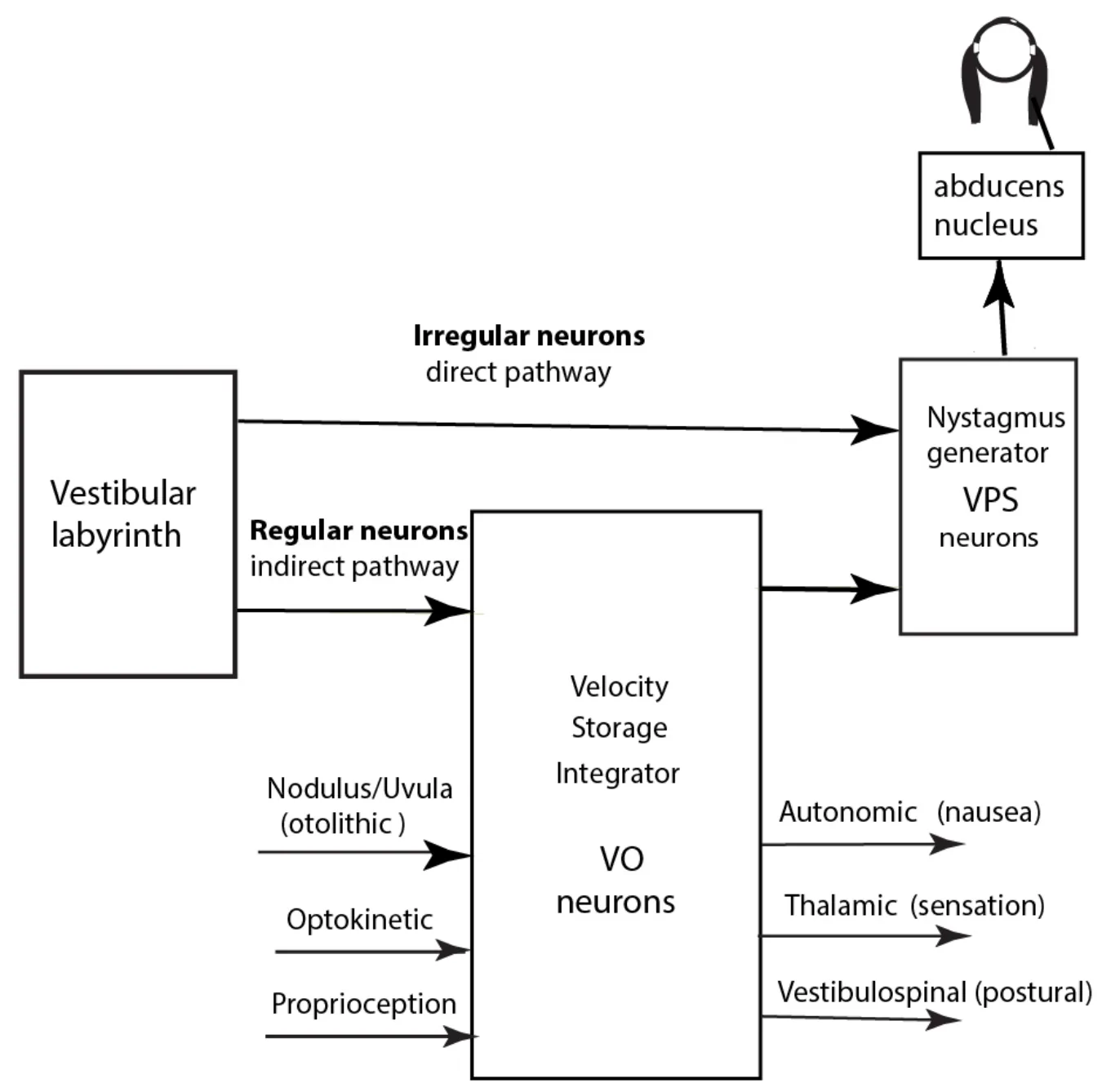
A simplified schematic illustration of the neural model I propose of the direct and indirect pathways, showing the velocity storage integrator, its inputs and outputs, based on evidence reviewed in [37]. Irregular primary neurons probably constitute the direct pathway, which projects from the labyrinth to the nystagmus generator, which drives abducens neurons to generate nystagmus. The indirect pathway is probably comprised of regular neurons which project to the brainstem velocity storage integrator (VO neurons), which then also project to the same nystagmus generator (VPS) neurons. The velocity storage integrator receives otolithic input from the nodulus and uvula and also input from optokinetic and proprioceptive sensory systems. The projections are to autonomic areas, the thalamus, and vestibulospinal projections.

It is VO neurons in the vestibular nuclei which comprise a major part of the velocity storage integrator [47] because the activity of the majority of VO neurons is closely related to the horizontal component of nystagmus and afternystagmus [47]. I propose these VO neurons receive predominantly regular afferent input. For recent reviews of the velocity storage integrator, see [48,49]. VO neuron firing rates are unaffected by alertness, and their firing rate declines with the time constant of velocity storage. This dissociation between VO and VPS neurons has the following consequences: VO neurons are the principal driving element of the velocity storage integrator, while in contrast VPS neurons provide the pathway from the vestibular system to the nystagmus generator subgroup. Most VPS neurons cease firing during drowsiness, and the quick phases of nystagmus are sensitive indicators of alertness. These vestibular-only (VO) neurons leave the VSI to drive the nystagmus generator subgroup (see Figure 6) [48]. So, both direct and indirect pathways result in nystagmus but with very different origins, and with the indirect pathway causing afternystagmus. Unlike caloric or post-rotational nystagmus, SVIN in patients with total unilateral vestibular loss causes little or no afternystagmus after stimulus offset, indicating that the velocity storage integrator is not contributing to the response and, in correspondence, these patients do not report vertigo at this time.

## 7. Afternystagmus

The absence of afternystagmus to mastoid vibration stimulation shows that the afferent input from irregular neurons either fails to “charge” the velocity storage integrator network, or bypasses it entirely, or is cancelled by the simultaneous otolithic activation by each cycle of the vibration stimulus. I discuss below that otolithic stimuli act to inhibit the VSI and suppress nystagmus by means of projections from the nodulus/uvula of the cerebellum to the vestibular nuclei. The absence of afternystagmus is unlike the response to angular acceleration of the canals, which does generate afternystagmus and vertigo. In short, vibration selectively drives the direct pathway and activates the nystagmus generator, but this selective drive is not sufficient to activate the VSI. That leads us to consider how afternystagmus is generated by the VSI and its significance in understanding the neural basis of vertigo.

## 8. A Brief Outline of Velocity Storage—The Probable Basis of Vertigo

The concept of the velocity storage integrator neural network was developed to explain the very long duration of afternystagmus following a step of angular velocity. Raphan and Cohen measured the duration of nystagmus after a sudden stop of yaw rotation in monkeys tested in darkness [50,51]. Such a stop deflects the cupula and activates both type I and type II hair cells of the horizontal semicircular canal and activates both irregular and regular afferents. After acceleration offset, the elastic restoring force within the cupula then returns the cupula to its resting position with a time constant of roughly 4 s [52]. This has been confirmed by recordings of primary afferent firing in such circumstances [53]. So, the neural activation of canal afferents returns to its resting discharge level within about 12 s after the stop [54]. However, Raphan and Cohen showed that the horizontal nystagmus that began with the cupula deflection at stopping continues for far longer than the primary afferent response. The nystagmus decays very slowly, with a time constant of roughly 18–20 s—three to four times longer than the cupula’s time constant [26,55].

So, the cupula alone cannot account for the prolonged afternystagmus—an additional central mechanism perseverates and prolongs the neural response. Raphan and Cohen proposed that this perseveration is due to the operation of a brainstem–cerebellar neural network which they called the velocity storage integrator—which had been “charged” by the afferent activation during the angular acceleration (much as a battery is charged). At the end of the acceleration, the activity in this perseverating neural network gradually ends and as it does so both the nystagmus and the subjective sensation of turning (the vertigo) decline together as we show below. So, this neural perseveration is the cause of the afternystagmus, and, as I suggest below, of vertigo.

## 9. Perseveration

**Figure 7:**
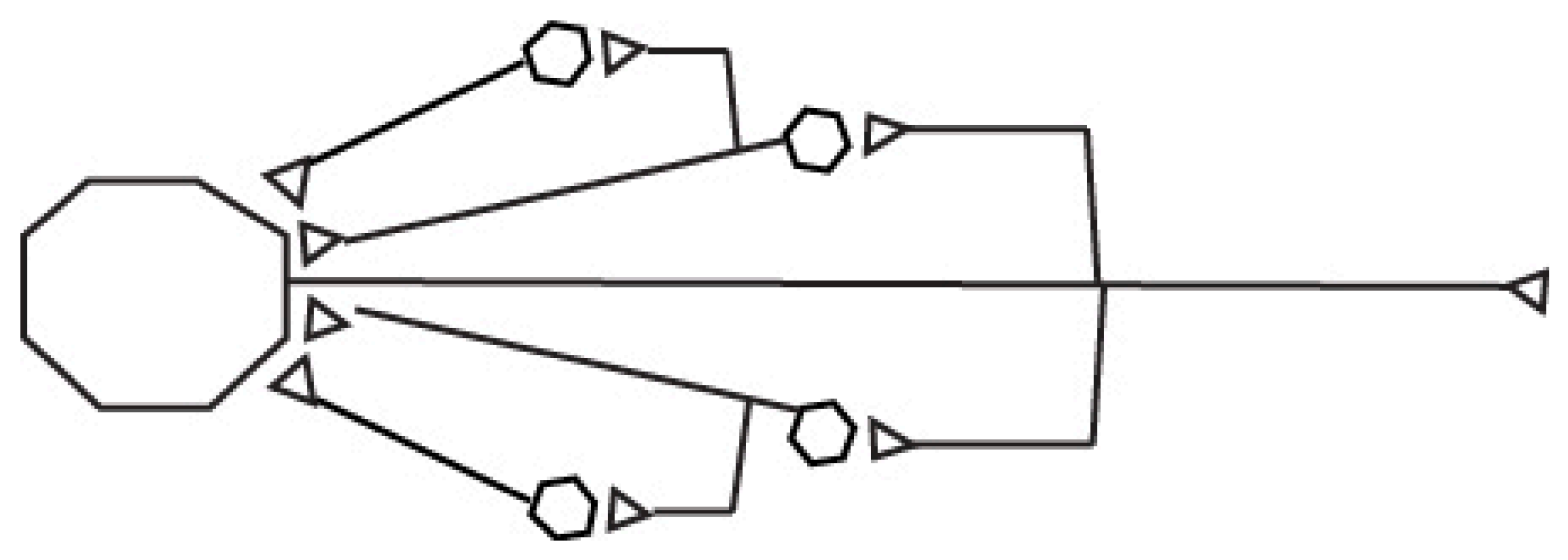
To show how a very simple neural network with positive feedback can result in perseveration. The axon from the neuron (large hexagon) projects to other neurons (small hexagons), which in turn feed back and excite the initial neuron.

How can a neural network prolong the neural response to an input? A version of a simple neural model proposed by Honrubia [56] Figure 7 shows how input can cause a neural network to keep perseverating or persisting until the response eventually decays.

Such a perseverating neural response has been shown by recording single neurons in the vestibular nucleus during afternystagmus [47]. The strongest evidence for a major role for the vestibular nuclei in the VSI is the evidence that the activity of some single neurons in the vestibular nucleus closely tracks the time course of afternystagmus decay [47]. Also, in response to localized electrical stimulation of the vestibular nuclei, Yokota and colleagues generated nystagmus that perseverated [57].

What could be the purpose of this perseverating neural activity? Raphan and Cohen proposed that the perseveration of the VSI network evolved to enhance the semicircular canals’ otherwise poor response to very low-frequency head movement: “By storing or integrating the signal coming from the cupula, the storage mechanism gives a more faithful representation of the very slow head velocity during slow head movement than the cupula can provide “ [50].

For many years, Cohen, Raphan, Waespe, and colleagues experimentally tested the factors that affect the VSI by measuring the afternystagmus and how various procedures disrupted afternystagmus. This work identified the extensive inputs visual, proprioceptive, and otolithic and the extensive outputs, which extend beyond vertigo to eye movements, postural control, autonomic function, and sensation [48]. A summary diagram of the major inputs to, and outputs from, the VSI is shown in Figure 6.

## 10. How Is Afternystagmus Related to Vertigo in Human Subjects?

**Figure 8:**
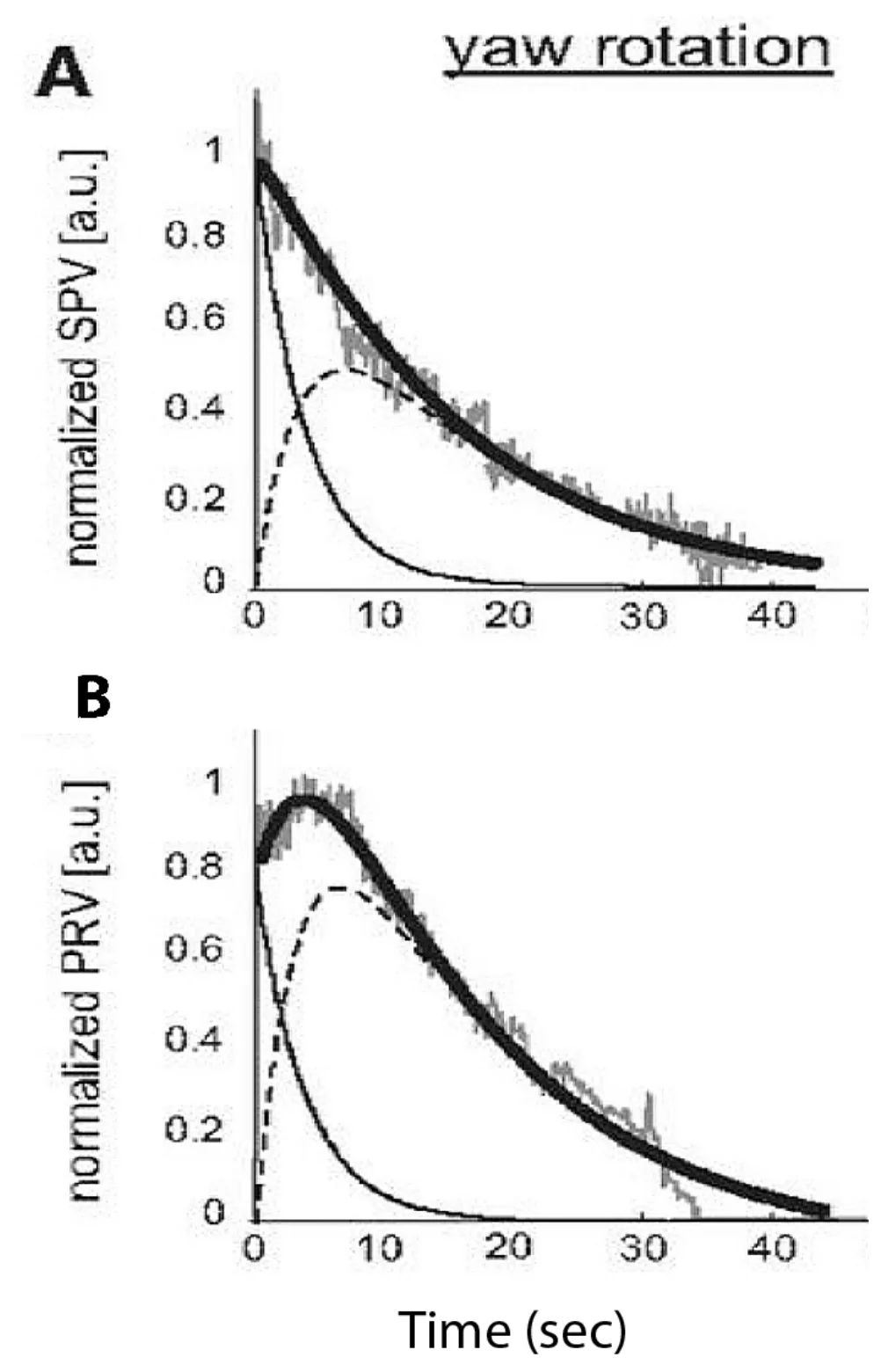
Plots of the decay of afternystagmus (**A**) and decay of perceived velocity (**B**) of a healthy human subject after an angular acceleration to show the close correspondence between the nystagmus decay and the decay of perceived velocity (each decay shown by thick lines). Since the person was stationary, the record in (**B**) shows the decay of the false sensation of rotation-vertigo. Reproduced from [58] with permission.

The research so far considered has been about nystagmus, because nystagmus is much easier to measure than vertigo. But the close relation between afternystagmus and vertigo is shown clearly by the following evidence. In human subjects, Bertolini and colleagues simultaneously tracked the decay of nystagmus after an abrupt step of angular velocity and simultaneously tracked the subject’s report of the decay of the subject’s sensation of turning—vertigo as I have defined it, because the subjects were stationary but still felt like they were turning. The evidence showed the two decay profiles matched closely (see Figure 8) [58,59].

Nystagmus decay after acceleration offset is slow, reflecting gradual discharge of the VSI, and the decay of vertigo followed a very similar time course (see Figure 8). Given the evidence that the VSI governs the decay of afternystagmus, this correspondence supports my contention that the VSI governs both nystagmus and the perception of self-rotation—reinforcing its role is not just in generating afternystagmus but in generating the conscious sensation of vertigo. As a result of this close correspondence, it is argued that procedures which affect afternystagmus decay in animals have a corresponding effect on vertigo.

The velocity storage integrator also receives input from the optokinetic system (Figure 6). The optokinetic input is a surrogate for vestibular input since many of the VSI results using vestibular input are paralleled closely by results using optokinetic stimulation. Large, slowly moving visual fields generate nystagmus and afternystagmus at the cessation of movement, together with sensations of vertigo comparable to those produced by vestibular stimulation, and there is long-standing evidence that optokinetic input feeds into the same velocity storage network [26]. Because optokinetic stimulation produces afternystagmus with the same characteristic decay as vestibular afternystagmus, this is taken as evidence for a projection from visual motion-processing structures into the VSI, alongside inputs from proprioception and other modalities.

## 11. What Stops the Perseveration of Neurons in the Velocity Storage Integrator?

Importantly, the rate of VSI decay is under otolithic control, via otolithic neurons that project to the nodulus and uvula of the cerebellum, and neurons from those structures in turn inhibit the VSI [60,61,62]. Many procedures that affect otolithic function alter the decay of afternystagmus and self-stability through this otolithic inhibition mediated by the nodulus-uvula. For example, a head tilt during nystagmus decay abolishes the afternystagmus—a phenomenon called tilt-dumping or otolithic suppression [63]. These procedures act to reduce or remove vertigo in human subjects and so deserve close attention for understanding patients’ experience and possibly alleviating vertigo. This otolithic control of the VSI is also demonstrated in monkeys by reduction of afternystagmus after electrical stimulation of the nodulus or uvula [62,64]. The nodulus and uvula exert inhibitory control over the VSI, and by implication, over vertigo. It seems that stored VSI activity is discharged by nodulus activity, which is triggered by otolithic input via a branch of primary otolithic afferents projecting to the nodulus/uvula region [50,55,60,65].

## 12. Outputs from the Velocity Storage Integrator

The outputs from the VSI are extensive (see Figure 6): as well as driving the nystagmus generator, the VSI projects to thalamic regions in order to generate the sensation of turning, to autonomic areas to trigger nausea and motion sickness, and to vestibulospinal projections for postural control [48].

Nystagmus and vertigo usually occur together, but not invariably: some subjects can suppress nystagmus while still experiencing pronounced vertigo, and other patients can have strong nystagmus without any vertigo [66]. The VSI is part of a neural circuit projecting to the thalamus. So, the perception of vertigo and self-motion is not governed by the VSI alone. Chiarovano reported patients who had vigorous caloric nystagmus but no accompanying sensation of rotation (i.e., no vertigo), which demonstrates this dissociation between nystagmus and vertigo directly. Because one output from the VSI projects to the thalamus to generate sensation, that output pathway (or its thalamic target) can evidently be affected—even suppressed—independently of the nystagmus-generating output. It seems more appropriate to consider the VSI as a key component in a complex neural circuit resulting in vertigo.

The model I have proposed here offers an insight into the relative contributions of regular and irregular primary afferents to nystagmus and vertigo. Some clinical tests probe the direct pathway (e.g., video head impulse testing, VEMPs) and give useful information about peripheral receptor function, but—because they do not engage the velocity storage integrator—they do not inform about vertigo or self-motion perception.

Vibration simultaneously activates irregular otolithic afferents, and otolithic input acts, via the nodulus/uvula pathway, to inhibit the VSI—so any nascent charging of the VSI by the canal input could be actively suppressed by the simultaneous otolithic input. It is also worth noting that large-area vibrators, such as those used in some prior studies [67] are likely to co-activate cervical afferents as well as activating vestibular receptors. Proprioceptive input is known to project to the velocity storage integrator [68]. This raises the possibility of testing these mechanisms directly—for instance, delivering a brief bone-conducted vibration stimulus during the decay phase of afternystagmus should activate irregular otolithic afferents projecting to the nodulus/uvula and, if the inhibitory hypothesis is correct, transiently suppress (“dump”) the ongoing afternystagmus and vertigo [55].

## 13. Discharge Regularity, Velocity Storage, and Vertigo

Taken together, the evidence supports my contention that SVIN reflects the activity of primary afferents with irregular resting discharge, while the usual route for charging the VSI—via caloric or rotational stimulation—is through afferents with regular resting discharge. On this account, caloric and rotational stimulation activate both regular and irregular afferents, engaging both the direct and indirect pathways (including the VSI), and so produce afternystagmus, whereas vibration, by selectively driving irregular afferents, does not result in afternystagmus.

In some instances, skull vibration can induce nystagmus and vertigo, for example in patients with semicircular canal dehiscence (SCD) [36,69] probably because the vibration stimulus induces fluid flow in the semicircular canals and thus cupula deflection and activation of regular neurons as well as irregular neurons [69].

This review extends ideas put forward in an earlier paper and highlights a new reason that the velocity storage integrator may not be functional during skull vibration stimulation. The reason is nodulus inhibition because of the unique simultaneous canal and otolith activation. This review has been brief out of necessity, and many of the important aspects of vertigo and the VSI are covered in detail in other reviews—for example, the fact that the VSI has to deal with turning in three dimensions. The very extensive modelling, the very significant input to the VSI from optokinetic stimulation, and its links to mal de embarquement are covered in reviews [47,48,70] to which the reader is referred.

I have emphasized the role of the nodulus throughout the paper. SVIN is a predominantly horizontal nystagmus. The major point of this paper is that the nodulus is important—it is the key new aspect in this work that vibration simultaneously activates both canal and otolith neurons and that such simultaneous activation by each cycle of the 100 Hz stimulus may be the reason that the afternystagmus is prevented whilst the nystagmus generator is still operational, but the velocity storage mechanism is disabled.

## 14. Limitations

The model I have proposed has a number of limitations. As I have noted, in primary afferents there is a wide range of the regularity of action potential firing at rest, but a useful simplification is to divide afferents into two major classes of afferent neurons according to the regularity of their resting discharge—regular and irregular. However, it is necessary to realize that I have categorized afferents into these two categories for the purpose of this paper, whereas there is a continuum of regularity [71].

Much of the physiological evidence reported here has come from studies of the physiology of the vestibular periphery of guinea pigs, and applying these data to human responses must be done with some caution. The above evidence is likely a reasonable approximation to data for human subjects since so much of the knowledge of the mechanics and physiology of the peripheral auditory system has derived from studies of the auditory system of guinea pigs.

As emphasized in the introductory sections, the human data about SVIN used here is derived from patients with total unilateral vestibular loss, since patients with partial vestibular loss show results which may be due to the residual vestibular function [72]. Patients with partial vestibular loss show somewhat different patterns of response compared to patients with total vestibular loss.

In outlining the model put forward in this paper, it may appear that the categories of afferents are clear-cut and categorical. But that appearance is due to the need to state the model unambiguously, and the variability of the published neural evidence means it is not categorical.

## 15. Conclusions and Clinical Value

Angular acceleration and caloric stimulation activate both irregular and regular canal afferents, producing nystagmus, vertigo, nausea, and postural responses. In contrast, mastoid vibration selectively activates canal and otolithic afferent neurons with irregular resting discharge. Mastoid vibration produces nystagmus, but there is no afternystagmus, so it is argued that in response to the vibration, the velocity storage integrator is not operational. Possible reasons are that this direct input of irregular afferents bypasses the velocity storage integrator, or that the vibration stimulates otoliths as well as canal afferents and that otolithic input may inhibit the VSI.

Bone-conducted vibration selectively activates irregular primary afferents and produces SVIN, which is a rapid clinical screening indicator of imbalance between the two vestibular systems. Because it probes the direct pathway, SVIN and the head impulse test provide complementary information to the information from slow rotation and caloric testing, which probe the indirect pathway and the velocity storage mechanism instead. Used together, these tests give a more complete picture of both peripheral vestibular receptor function and the central mechanisms underlying vertigo.

The absence of afternystagmus following 100 Hz vibration in patients with total unilateral vestibular loss indicates that, although vibration produces nystagmus, there is no afternystagmus. So, vibration-induced nystagmus occurs without afternystagmus. This dissociation offers a valuable window into the relative contributions of regular and irregular afferents to nystagmus and vertigo: standard clinical tests that probe the direct pathway (video head impulse testing, VEMPs) give useful information about peripheral receptor function, but—because they do not engage the VSI—they do not inform directly about vertigo or self-motion perception.

## Data Availability

No new data were created or analyzed in this study. Data sharing is not applicable to this article.

## Abbreviations

The following abbreviations are used in this manuscript:

SCD	semicircular canal dehiscence
SVIN	skull vibration-induced nystagmus
VEMP	vestibular evoked myogenic potentials
VSI	velocity storage integrator
